# Comparative effects of curcumin versus nano-curcumin on histological, immunohistochemical expression, histomorphometric, and biochemical changes to pancreatic beta cells and lipid profile of streptozocin induced diabetes in male Sprague–Dawley rats

**DOI:** 10.1007/s11356-023-26260-6

**Published:** 2023-03-18

**Authors:** Mohamed R. Metawea, Heba M. A. Abdelrazek, Heba Nageh Gad El-Hak, Mona M. Moghazee, Ohoud M. Marie

**Affiliations:** 1 Chemistry Department, Faculty of Science, Suez Canal University, Ismailia, 41522 Egypt; 2 Physiology Department, Faculty of Veterinary Medicine, Suez Canal University, Ismailia, 41522 Egypt; 3 Zoology Department, Faculty of Science, Suez Canal University, Ismailia, 41522 Egypt; 4 Genetics Department, Faculty of Agriculture, Ain Shams University, Cairo, 11241 Egypt

**Keywords:** Caspase-3, Curcumin, Diabetes, Immunohistochemical expression, Nano-curcumin, Oxidative stress, Pancreas

## Abstract

Diabetes mellitus is a worldwide problem characterized by hyperglycemia as well as the damage of the microscopic structure of the beta cells of Langerhans pancreatic islets. In the present study, the histological, immunohistochemical, morphometric, and biochemical alterations to pancreatic beta cells in streptozocin (STZ)-induced diabetes were assessed in rats treated with curcumin (CU) (100 mg/kg/day) or nano-curcumin (nCU) (100 mg/kg/day) for 1 month. Twenty-four adult male Wistar albino rats were distributed into four groups: the nondiabetic control group, the diabetic untreated group, and two diabetic groups treated with CU or nCUR, respectively. Blood glucose, serum insulin levels, and lipid profile were measured. The pancreatic tissues were collected and processed into paraffin sections for histological and immunohistochemical examination, oxidative stress markers, and real-time PCR expression for pancreatic and duodenal homeobox 1 (PDX1). The insulin expression in beta cells was assessed using immunohistochemistry. Morphometrically, the percentage area of anti-insulin antibody reaction and the percentage area of islet cells were determined. STZ-induced deteriorating alteration in beta cells led to declines in the number of functioning beta cells and insulin immunoreactivity. In STZ-treated rats, CU and nCUR significantly reduced blood glucose concentration while increasing blood insulin level. It also caused a significant increase in the number of immunoreactive beta cells to the insulin expression and significant reduction of the immunoreactive beta cells to the caspase-3 expression. In conclusion, CU and nCUR could have a therapeutic role in the biochemical and microscopic changes in pancreatic beta cells in diabetes-induced rats through STZ administration with more bio-efficacy of nCUR.

## Introduction

Diabetes mellitus is a diverse metabolic disorder featured by the incidence of hyperglycemia based on diminishing of insulin secretion, defective insulin action, or both (Lin and Sun 2010). Diabetic glucose variation in turn affect the magnitude of oxidative stress (Lucchesi et al. 2013). Oxidative stress has been suggested as a potential mechanism for diabetes-associated tissue as well as other systemic problems (Sczepanik et al. 2020; Abbasi et al. 2022). Consequently, it is reasonable to conclude that the treatment of diabetes and its consequences depends on the replenishment of insulin-producing pancreatic β-cells (Bonner-Weir and Weir 2005), thereby addressing the limitations of present therapeutic approaches. Recently, it is established that both types of diabetes mellitus, type I and II, have an impact on the number of β-cells and insulin secretion (Díaz-Villaseñor et al. 2006). This finding has motivated renewed attention in targeting the pancreatic β-cell. The understanding of the pancreatic β-cell holds promise for enhancing glycaemia management and maybe slowing the development of diabetes complications (Garber 2011).

Curcumin (CUR) is a natural polyphenol, with two o-methoxy phenolic groups, one enone moiety, and an α, β-unsaturated diketone group (Indira Priyadarsini 2013). It has keto-enol tautomerism (Rege et al. 2019). CUR decreases blood glucose as well as glycosylated hemoglobin levels and prohibited weight loss (Jain et al. 2009). It was also suggested to decrease numerous other complications related to diabetes as fatty liver, diabetic neuropathy, diabetic nephropathy, vascular diseases, musculoskeletal diseases, and islet viability (Kunwar and Priyadarsini 2016; Gad El-Hak and Mobarak 2020). Due to its potential health advantages, researchers are paying great attention to CUR, a phytochemical found in the spice turmeric (Khorasani et al. 2019). Though CUR has several pharmacologic effects, its weak oral bioavailability, low digestive system absorption, and tendency to be excreted instead of dispersed consistently reduce its therapeutic effectiveness (Abu-Taweel et al. 2020).Hence, it is valuable to afford approaches that can enhance the solubility of functional phytoconstituents that have a poor solubility in water and keep them safe until they reach the desired destination in the body. Several approaches have been established to increase the efficiency of CUR. Liposomal CUR, CUR phospholipid complex, CUR nano-particles, and CUR nano-capsules are examples of these methods (Anwar et al. 2021).

In compared to normal CUR, nano-curcumin (nCUR) is recommended as the ideal alternative to be utilized as a treatment. It has a bigger surface area and is more bioavailable, so it can penetrate organs that CUR cannot (Karthikeyan et al. 2020). High plasma concentration, minimal toxicity, and development of an immunological response are all provided by nCUR exosomes (Moballegh Nasery et al. 2020). They can be useful against venom activities, protozoal and microbial contamination, inflammatory responses, angiogenesis, and tumor suppression due to the presence of exosomes, in addition to their anti-aging and anti-oxidant characteristics (Moballegh Nasery et al. 2020).

Based on these facts, the current study was designed to investigate histological, immunohistochemical expression as well as oxidative stress variations in the pancreatic tissue of STZ-induced diabetic rats after CUR and nCUR treatment. The later effect was achieved by serum levels of fasting blood glucose, insulin, and lipid profile estimation. Also, pancreatic lipid peroxidation malondialdehyde (MDA) and antioxidants such as superoxide dismutase (SOD) and glutathione (GSH) were determined. Real-time polymerase chain reaction (Rt-PCR) for pancreatic PDX1 genes was detected.

## Materials and methods

### Chemicals

Streptozotocin (STZ) was purchased from MP Biomedicals, LLC, USA; acetic acid, chitosan derived from shrimp shells in a 75% (de-acetylated) concentration (de-acetylated), was got from LOBA CHEMIE PVT.LTD, India; CUR was purchased from LANXESS AG headquarters, India, with dimethyl sulfoxide (DMSO) and deionized water. Sodium tripolyphosphate (TPP) was obtained from LANXESS AG Headquarters, India.

#### Preparation of CUR nanoparticles (CUR-NPs)

According to Aktaş et al. (2005), the mechanism behind the formation of nCUR was an ionic interaction between the positively charged amino groups of chitosan and the negatively charged groups of his TPP. Briefly, to prepare a solution of chitosan, 1 g of chitosan was dissolved in 200 mL of 2.5% acetic acid with stirring with a magnetic stirrer. A solution of sodium tripolyphosphate (TPP) was prepared by dissolving 0.66 g of TPP in 50 mL of distilled water. Alike, a solution of curcumin was prepared by dissolving 1 g of curcumin in 40 mL of DMSO and magnetically stirring for 30 min. To synthesize nCUR, the curcumin solution was dropped into the chitosan solution in the beaker and stirred for 15 min to evenly disperse the curcumin in the chitosan solution. This was followed by the dropwise addition of sodium tripolyphosphate (50 mL) and stirring continuously with a magnetic stirrer for 45 min. The entire suspension was centrifuged at 11,000 rpm for 30 min. Then, the nCUR-containing pellet was lyophilized and stored in a 4 °C refrigerator for further use.

#### Characterization of the nanoparticles

##### Transmission electron microscopy (TEM)

TEM analysis was applied at NanoTech, Egypt to identify the surface morphology of the prepared nCUR. One drop of nanosuspension was allowed to be adhered to the paraffin sheet by covering with a copper grid. After removing the residual solution, the samples were air dried and examined under a TEM (Serra-Maia et al. 2021).

##### Determination of the entrapment efficiency (EE) and loading capacity (LC)

Encapsulation efficiency is the ability of a drug to be efficiently encapsulated and indicates the percentage of successfully encapsulated drug. The loading capacity is the total amount of drug trapped per total weight of nanoparticles. The free amount of curcumin in the supernatant, left after preparation of loaded nanoparticles, was estimated using UV–Vis spectroscopy. The following equations were used after three measurements to calculate the EE and LC of the prepared nanoparticles (Ahmad et al. 2016).

$$\mathrm{EE}\;(\%)=(\mathrm t\mathrm o\mathrm t\mathrm a\mathrm l\;\mathrm d\mathrm r\mathrm u\mathrm g-\mathrm f\mathrm r\mathrm e\mathrm e\;\mathrm d\mathrm r\mathrm u\mathrm g)/\mathrm t\mathrm o\mathrm t\mathrm a\mathrm l\;\mathrm d\mathrm r\mathrm u\mathrm g\times100$$

$$\mathrm{LC}\;(\%)=(\mathrm t\mathrm o\mathrm t\mathrm a\mathrm l\;\mathrm d\mathrm r\mathrm u\mathrm g-\mathrm f\mathrm r\mathrm e\mathrm e\;\mathrm d\mathrm r\mathrm u\mathrm g)/\mathrm w\mathrm e\mathrm i\mathrm g\mathrm h\mathrm t\;\mathrm o\mathrm f\;\mathrm N\mathrm P\mathrm s\times100$$

### Animals

Twenty male Sprague–Dawley rats weighing 180–200 g were obtained from the animal facility of the National Research Center of Egypt (NRC). They were housed in metal cages (5 animals/cage) in the Laboratory Animal House of the Faculty of Veterinary Medicine, Suez Canal University, Egypt. The rats were maintained under a natural daylight cycle at a temperature of 25 ± 2 °C and were allowed to acclimatize for 2 weeks before the beginning of animal experiments. Rats were exposed to free water and normal rodent chow. Recommendations for ethical care and treatment of animals followed the regulations of the National Research Center (NRC) Ethics Committee. The study design was approved by the Faculty of Science Ethics Committee (approval no. REC55/2020).

### Experimental design

After 14 days of acclimation, the animals were divided into 4 equal groups (5 rats each) as follows:

1. Control group: normal non-diabetic rats
2. Diab group: STZ-induced diabetic rats
3. Diab + CUR group: STZ-induced diabetic rats that received a daily oral dose of 100 mg/kg/day of CUR dissolved in carboxymethylcellulose (CMC) 1:10 (w/v) for 1 month (Shamsi-Goushki et al. 2020)
4. Diab + nCUR group: STZ-induced diabetic rats given a daily oral dose of 100 mg/kg/day of nCUR dissolved in CMC-1:10 (w/v) for 1 month (Shamsi-Goushki et al. 2020)

### Induction of diabetes according to Masiello et al. (1998)

Streptozotocin was freshly prepared at a dose of 65 mg/kg body weight dissolved in 0.1 M citrate buffer (pH 4.5) at a volume of 1 mL/kg body weight and injected intraperitoneally (IP) within 5 min. To confirm the presence of diabetes, fasting blood sugar (FBS) was measured after 3 days using reagent strips (Accu-Chek®, Roche, Germany). Rats with a blood glucose range of 250 mg/dL were considered diabetic.

### Sampling

At the end of the experiment, all rats were fasted for 12 h; then, blood was drained and collected into two tubes under tetrahydrofuran inhalation anesthesia. The first tube contained sodium fluoride to measure levels of FBS. The second tube was a simple tube to separate serum for determining lipid profiling and insulin. Two tubes were centrifuged at 3000 rpm for 10 min using a cooled centrifuge to collect plasma and sera. Sera were stored in a − 80 °C freezer. Animals were euthanized and dissected to obtain the pancreas of each rat. After weighing them, each one was divided into three parts. The first part of the pancreas was kept at − 80 °C for further homogenization, and the second part was immersed in the RNA latter and kept at − 80 °C. The third portion of the pancreas was immersed in 10% neutral buffered formalin for histopathological and immunohistochemical examination.

### Body weight

Experimental rats were weighed weekly throughout the experimental period. Weight gain per week was also determined by subtracting two consecutive body weights.

### Blood glucose, insulin levels, and HOMA-IR

Using the technique outlined by Aguilar et al. (2001), fluorinated plasma samples were exposed to glucose measurement using a diagnosis reagent kit (SPINREACT, Spain) (2001). According to the supplied manufacturer’s protocol, the levels of insulin in sera were determined using a commercial rat enzyme-linked immunosorbent assay (ELISA) kit from SPI-BIO in France. The homeostasis model assessment approach, HOMA-IR, was used to estimate insulin resistance. Its calculation is as follows: plasma glucose (mg/dL) × fasting plasma insulin (IU mg/L in the fasting state divided by 405) (Ghiasi et al. 2015).

### Lipid profile

According to Saggu et al. (2014), enzymatic colorimetric kits (SPINREACT, Spain) were used to assess the serum levels of total cholesterol (TC), high-density lipoprotein cholesterol (HDL-c), low-density lipoprotein cholesterol (LDL-c), and triglycerides (TG).

### Lipid peroxidation and antioxidants

Pancreatic homogenate was prepared by homogenization of 1 g of tissue in 4.5-mL cold potassium buffer pH 7.4. Centrifugation was at 13,000 × g for 10 min at 4 °C in cooling centrifuge (Brahmer 2012). After collection, the supernatant was stored at − 80 °C until use. Malondialdehyde (MDA), as lipid peroxidation biomarker level, was estimated by ELISA kit (Lifespan Biosciences, USA). Pancreatic homogenate-reduced glutathione (GSH) level was estimated by ELISA (Shanghai Blue Gene Biotech, China). Pancreatic homogenate superoxide dismutase (SOD) level was estimated by ELISA kit (Elabscience, USA).

### Histopathology and histomorphometry evaluation for the pancreas

Pancreatic tissue that had been formalin-fixed was placed in paraffin wax after being dehydrated in an ethyl alcohol gradient (70, 80, 90, 95, and 99.9%). According to Feldman and Wolfe (2014), several 5-µm slices were cut and stained with hematoxylin and eosin (H&E). The slides were investigated blindly. By measuring the area of four islets in each part of one rat from each group, the average area and diameter of the islets were calculated.

### Immunohistochemical expression

For the immunohistochemical detection of insulin and caspase-3 (an indicator for apoptosis), the other sections were attached on positively charged slides. A rabbit anti-cleavage caspase-3 monoclonal antibody (Cell Signaling Technology, Boston, MA, USA, Cat. No. 9661 1:20 dilution) and a polyclonal guinea pig anti-insulin antibody were employed to be incubated with the slides (N1542, Dako, Carpinteria, CA, USA 1:100 dilution). Generally, the dilution was done using standard immunohistochemistry techniques overnight at 4 °C, as described by Gad El-Hak et al. (2021) and Abunasef et al. (2014a). Using a light microscope and an associated image capture and processing device, sections were imaged. The findings of the quantification were reported as the mean area of immunopositive cells per square millimetre in islets using the Fiji/ImageJ software. Five islets were randomly chosen from five paraffin blocks from five microscopic fields among five rats in each group.

#### RNA isolation, reverse transcription, and quantitative real-time quantitative PCR (qRT-qPCR)

Using the GenejET RNA Purification Kit (Thermo Scientific, USA), total RNA was extracted from the pancreas of each rat as directed by the manufacturer. Thermo Scientific RevertAid™ M-MuLV Reverse Transcriptase was used to reverse-transcribe two micrograms of isolated RNA in a 20-L reaction volume. Maxima SYBR Green/ROX qPCR Master Mix (2X) (Thermo Scientific, USA) kit was used for quantitative real-time polymerase chain reaction (RT-qPCR) utilizing a light cycler device (Applied CFX96 Touch System, Bio-RAD). Thermal cycling conditions included a 10-min initial activation step at 95 °C, followed by 40 cycles, one of which included a 15-s denaturation step. A single PCR result from each primer was validated by analyzing melting curves. Beta actin, Pdx1, and HNF4A primers were manufactured by the Macrogen Company in Germany. The complete list of primer sequences used in this study can be found in Table 1. The fold change in gene expression was calculated using comparative quantitation analysis (2–Δ(ΔCt) technique) and was adjusted to *β-*actin as a housekeeping gene.

**Table 1:** Primer sequences of target genes that were used for real-time PCR

No	Gene	Rotation	Sequence	Amplicon size
	*PDX1*	F	ACCCGTACAGCCTACACTCG	198
		R	CGTTGTCCCGCTACTACGTT	
	*β-actin*	F	ACAACCTTCTTGCAGCTCCTC	200
		R	CTGACCCATACCCACCATCAC	

*F*, forward, *R*, reverse primer

## Statistical analysis

The data was found to be normal after normality testing via Shapiro–Wilk test. The latest version of GraphPad Prism for Windows was used to evaluate the results. Mean ± SE (*n* = 5) was used to express the results. The Tukey test was used to assess group differences after a one-way ANOVA. *P*-values under 0.05 were regarded as significant.

## Results

### Transmission electron microscopy (TEM)

**Fig. 1:**
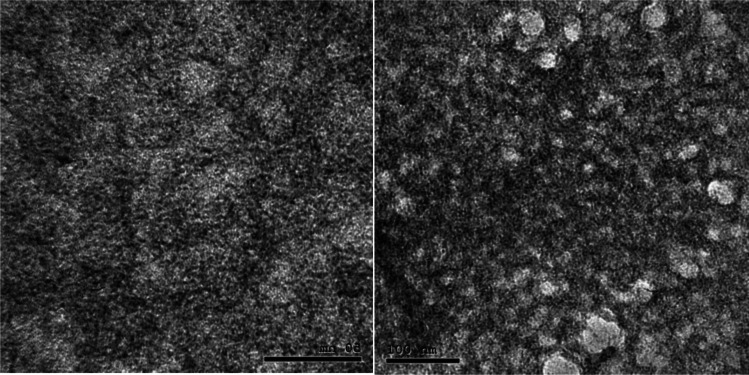
TEM for nCUR

The TEM technique was used to determine the nanoparticles’ size and surface form. nCUR had a mean size of 39.9 nm and was spherical (Fig. 1).

### Determination of the entrapment efficiency (EE) and loading capacity (LC)

Discovered 43.1% w/s was higher efficacy of drug deviation (EE %) for NCUR. However, the loading capacity (LC%) was 13.9% w/w.

#### Body weight

When compared to the nondiabetic control group, the final body weights of the STZ-induced diabetic group rats decreased significantly (*P* < *0.05*). However, when CUR and nCUR were administered to diabetic rats, their final body weights rose (*P* < 0.05) in contrast to diabetic non-treated rats (Table 2).

**Table 2:** Effect of curcumin (CUR) and nano-curcumin (nCUR) on initial and final weight of diabetic rats

Body weight (g)	Control	Diab	Diab + CUR	Diab + nCUR
Initial weight	187.8 ± 3.34	187.2 ± 4.27	189.6 ± 7.02	189.4 ± 9.35
Final weight	222.00^a^ ± 8.60	171.40^c^ ± 10.83	195.2^b^ ± 2.82	194.4^b^ ± 20.60

Each value represents the mean ± SE (*n* = 5). Values with different superscript letters within the same row are significantly different at *P* < 0.05. Diab, STZ-induced diabetic rats; Diab + CUR, STZ-induced diabetic rats that were given a daily oral dose of 100 mg/kg/day of curcumin were dissolved in carboxymethylcellulose (CMC) 1:10 (w/v); Diab + nCUR, STZ-induced diabetic rats that were given a daily oral dose of 100 mg/kg/day of nano-curcumin were dissolved in carboxymethylcellulose (CMC) 1:10 (w/v)

#### Fasting blood glucose value (FBG), insulin level, and HOMA-IR

In comparison to the control group, experimental rats receiving a single IP dosage of STZ showed a significant (*P* < 0.05) increase in FBG levels. In comparison to the diabetic non-treated group, CUR and nCUR administration to diabetic rats for one month resulted in a significant (*P* < *0.05*) decrease in the levels of FBG. The STZ diabetic group's insulin level showed a substantial (*P* < *0.05*) decrease compared to the control group. In contrast, diabetic rats treated for a month with CUR and nCUR had considerably (*P* < 0.05) higher insulin levels than diabetic rats who were not treated. Significant (*P* < *0.05*) insulin level promotion was seen in the nCUR compared to the CUR. In comparison to the control group, the STZ diabetic rats displayed a significant (*P* < 0.05) increase in HOMA-IR. After one month of CUR and nCUR treatment for diabetic rats, HOMA-IR has been found to decrease to levels similar to those of the control group (Table 3).

**Table 3:** Effect of curcumin (CUR) and nano-curcumin (nCUR) on fasting blood glucose value, insulin level, HOMA-IR, and lipid profile of diabetic rats

	Control	Diab	Diab + CUR	Diab + nCUR
FBG (mg/dL)	86.80^b^ ± 4.25	627.4^a^ ± 23.15	166.2^b^ ± 42.31	157.6^b^ ± 19.85
Insulin (pmol/L)	71.41^a^ ± 0.47	35.41^d^ ± 0.45	44.48^c^ ± 0.48	60.77^b^ ± 0.43
HOMA-IR	15.26^b^ ± 0.72	54.88^a^ ± 2.27	18.15^b^ ± 4.51	23.60^b^ ± 2.88
HDL-c (mg/dL)	53.20^a^ ± 0.86	41.00^b^ ± 1.52	54.40^a^ ± 1.81	56.20^a^ ± 3.94
LDL-c (mg/dL)	24.40^c^ ± 1.54	50.00^a^ ± 4.62	38.20^b^ ± 3.69	34.80^b^ ± 1.72
TG (mg/dL)	54.20^c^ ± 2.35	86.80^a^ ± 4.28	72.20^b^ ± 4.79	62.60^bc^ ± 1.36
TC (mg/dL)	88.20^b^ ± 2.13	110.6^a^ ± 2.04	107.4^a^ ± 3.44	105.4^a^ ± 1.63

Each value represents the mean ± SE (*n* = 5). Values with different superscript letters within the same row are significantly different at *P* < 0.05. *FBG*, fasting blood glucose; *HDL-c*, high-density lipoprotein cholesterol; *LDL-c*, low-density lipoprotein cholesterol; *TC*, total cholesterol; and *TG*, triglycerides; Diab, STZ-induced diabetic rats; Diab + CUR, STZ-induced diabetic rats that were given a daily oral dose of 100 mg/kg/day of curcumin were dissolved in carboxymethylcellulose (CMC) 1:10 (w/v); Diab + nCUR, STZ-induced diabetic rats that were given a daily oral dose of 100 mg/kg/day of nano-curcumin were dissolved in carboxymethylcellulose (CMC) 1:10 (w/v)

#### Lipid profile

Diabetic rats treated for a month with CUR and nCUR had considerably (*P* < 0.05) higher insulin levels than diabetic rats that were not treated. Significant (*P* < *0.05*) insulin level promotion was found in the nCUR compared to the CUR. In comparison to the control group, the STZ diabetic rats displayed a significant (*P* < 0.05) increase in HOMA-IR. After one month of CUR and nCUR treatment for diabetic rats, HOMA-IR has been found to decrease to levels similar to those of the control group (Table 3).

#### Lipid peroxidation and antioxidants

Current data revealed that diabetic rats induced with STZ had significantly lower pancreatic SOD activity and GSH concentration than control rats (*P* < 0.05). When compared to the diabetic non-treated group, the pancreatic SOD and GSH activities of the diabetic rats receiving CUR and nCUR for a month were considerably (*P* < 0.05) higher. In comparison to CUR, administration of nCUR resulted in a substantial (*P* < 0.05) improvement in GSH content and SOD activity. Pancreatic MDA levels significantly increased (*P* < 0.05) in the Diab group compared to the control group. When compared to diabetic rats that weren't given CUR or nCUR for a month, there was a significant (*P* < 0.05) reduction in the amount of MDA in the pancreas (Table 4).

**Table 4:** Effect of curcumin (CUR) and nano-curcumin (nCUR) on lipid peroxidation and anti-oxidants of diabetic rats

Parameter	Control	Diab	Diab + CUR	Diab + nCUR
SOD	58.66^a^ ± 5.32	10.40^d^ ± 1.12	21.38^c^ ± 1.24	43.14^b^ ± 2.18
GSH	71.34^a^ ± 3.96	11.56^d^ ± 1.01	22.96^c^ ± 1.83	44.00^b^ ± 3.52
MDA	9.16^c^ ± 0.97	47.48^a^ ± 4.15	26.50^b^ ± 1.53	17.74^b^ ± 0.88

Each value represents the mean ± SE (*n* = 5). Values with different superscript letters within the same row are significantly different at *P* < 0.05. Diab, STZ-induced diabetic rats; Diab + CUR, STZ-induced diabetic rats were given a daily oral dose of 100 mg/kg/day of curcumin were dissolved in carboxymethylcellulose (CMC) 1:10 (w/v); Diab + nCUR, STZ-induced diabetic rats that were given a daily oral dose of 100 mg/kg/day of nanocurcumin were dissolved in carboxymethylcellulose (CMC) 1:10 (w/v)

#### PDX1 gene

**Fig. 2:**
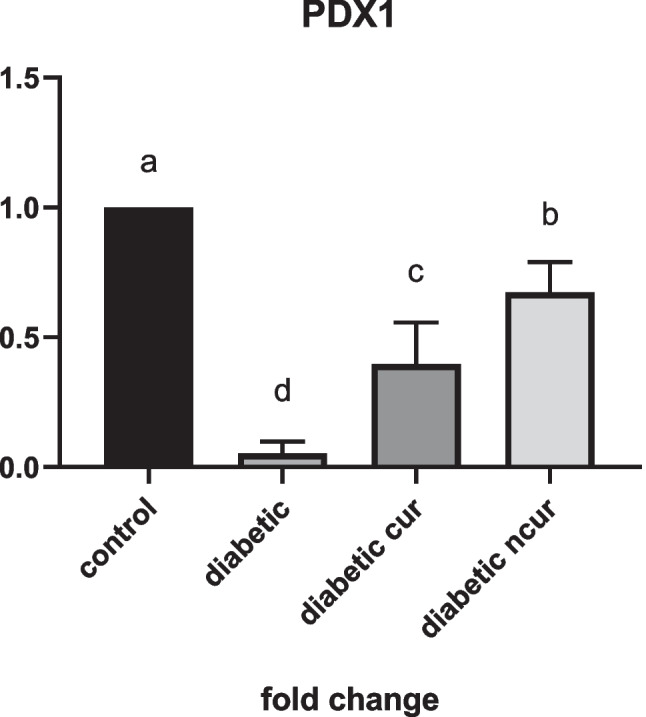
(a–d) Effect of curcumin (CUR) and nano-curcumin (nCUR) on for pancreatic and duodenal homeobox 1 (PDX1) of diabetic rats. Data was expressed as mean ± SE. Statistical significance was evaluated by one-way ANOVA followed by Tukey test at *P* < 0.05. The mean values with different superscript letters on the bars were significantly different at *P* < 0.05

The current findings demonstrated a significantly lower fold change of PDX1 expression in diabetic rats as compared to the control group (*P* < 0.05). PDX1 expression significantly increased after receiving CUR and nCUR treatment compared to diabetic rats. Significant (*P* < 0.05) increases in fold change were seen in the nCUR-treated group relative to the CUR treated group (Fig. 2).

## Histopathology and histomorphometry evaluation for the pancreas

The histopathology of rat pancreas of control is shown in Fig. 2 (a). The microscopic investigation of their pancreatic slices showed the regular appearance of islets of Langerhans surrounded with regular acinar cells. The islets of Langerhans looked as unencapsulated pale stained rounded or oval areas inside the pancreatic lobules, which were made of groups of cells arranged in irregular, branching, and anastomosing cords separated by blood capillaries. However, the Diab rats showed atrophic, shrunken, and degenerated islet cells with small vacuoles that were detected in the acinar cells. An apparent decrease and atrophy in the size of islets is observed in Fig. 2 (b). On the other hand, Diab + CUR and Diab + nCUR groups represented an evidence of cellular restoration among the islets of Langerhans with limited degenerated cells of the islets that were detected (Fig. 2 (c, d)). The islets revealed nearly normal outlines and almost normal cell histology.

The histomorphometry evaluation for the pancreas indicated the percentage area of β-cells, and the diameter of the islets was significantly (*P* < 0.05) reduced in diabetic rats compared to control group. However, both the treated diabetic groups with CUR and nCUR showed significant (*P* < 0.05) increase in all morphometric parameters, as compared with the untreated diabetic group (Fig. 2e, f).

**Fig. 3:**
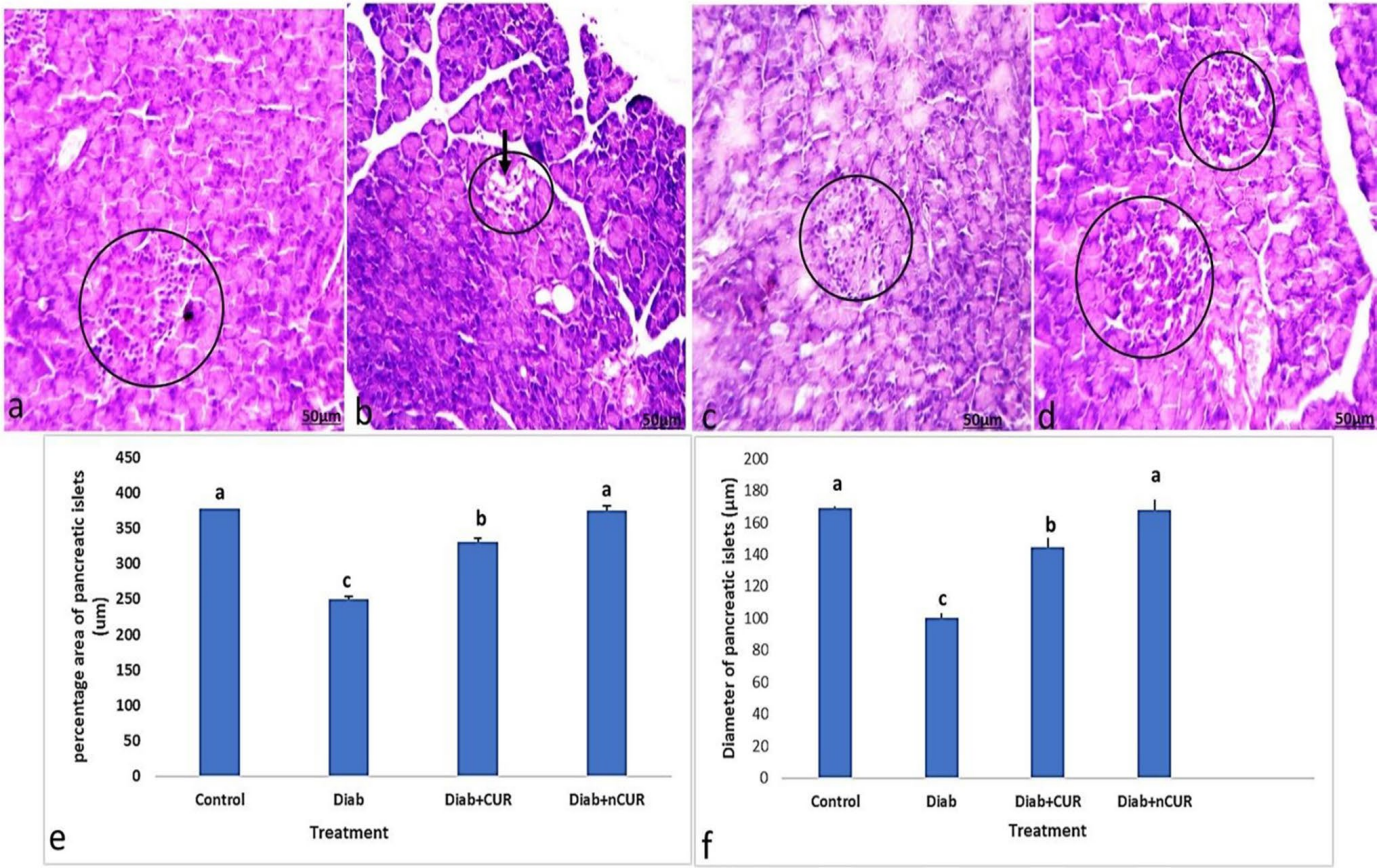
**a**–**d** Photomicrographs of sections of the pancreas stained by H&E. **a** Section from control group showed normal alpha-cells and beta cells (circle). **b** Section from pancreas of Diab rat showed atrophy and cytoplasmic degenerative changes (arrow) in most islet cells, especially in center of the islet (circle). **c** Section from pancreas of (Diab + CUR**)** STZ-induced diabetic rats that were given a daily oral dose of 100 mg/kg/day of curcumin showed very similar morphology to the control group. **d** Section from rat of (Diab + nCUR**)** STZ-induced diabetic rats that were given a daily oral dose of 100 mg/kg/day of nano-curcumin showed nearly regular outline of an islet with apparently normal appearance of most cells (H&E staining, × 100). **e**, **f** Histomorphometric measurements showed changes in the mean values of the following: **e** percentage area of pancreatic islets cells (%); **f** diameter of islet cells. Data was expressed as mean ± SE. Statistical significance was evaluated by one-way ANOVA followed by Tukey test at *P* < 0.05. The mean values with different superscript letters on the bars were significantly different at *P* < 0.05. Diab, STZ-induced diabetic rats; Diab + CUR, STZ-induced diabetic rats that were given a daily oral dose of 100 mg/kg/day of curcumin were dissolved in carboxymethylcellulose (CMC) 1:10 (w/v); Diab + nCUR, STZ-induced diabetic rats that were given a daily oral dose of 100 mg/kg/day of nano-curcumin were dissolved in CMC 1:10 (w/v)

The percentage area of β-cells and the width of the islets were considerably (*P* < 0.05) smaller in diabetic rats compared to the control group, according to the pancreas’ histomorphometry examination. However, compared to the untreated diabetes group, both the diabetic groups treated with CUR and nCUR showed substantial (*P* < 0.05) increases in all morphometric measures. In comparison to Diab + CUR (Fig. 3), Diab + nCUR significantly improved pancreatic morphometric characteristics (Fig. 3e, f).

## Immunohistochemistry of rat’s pancreas

**Fig. 4:**
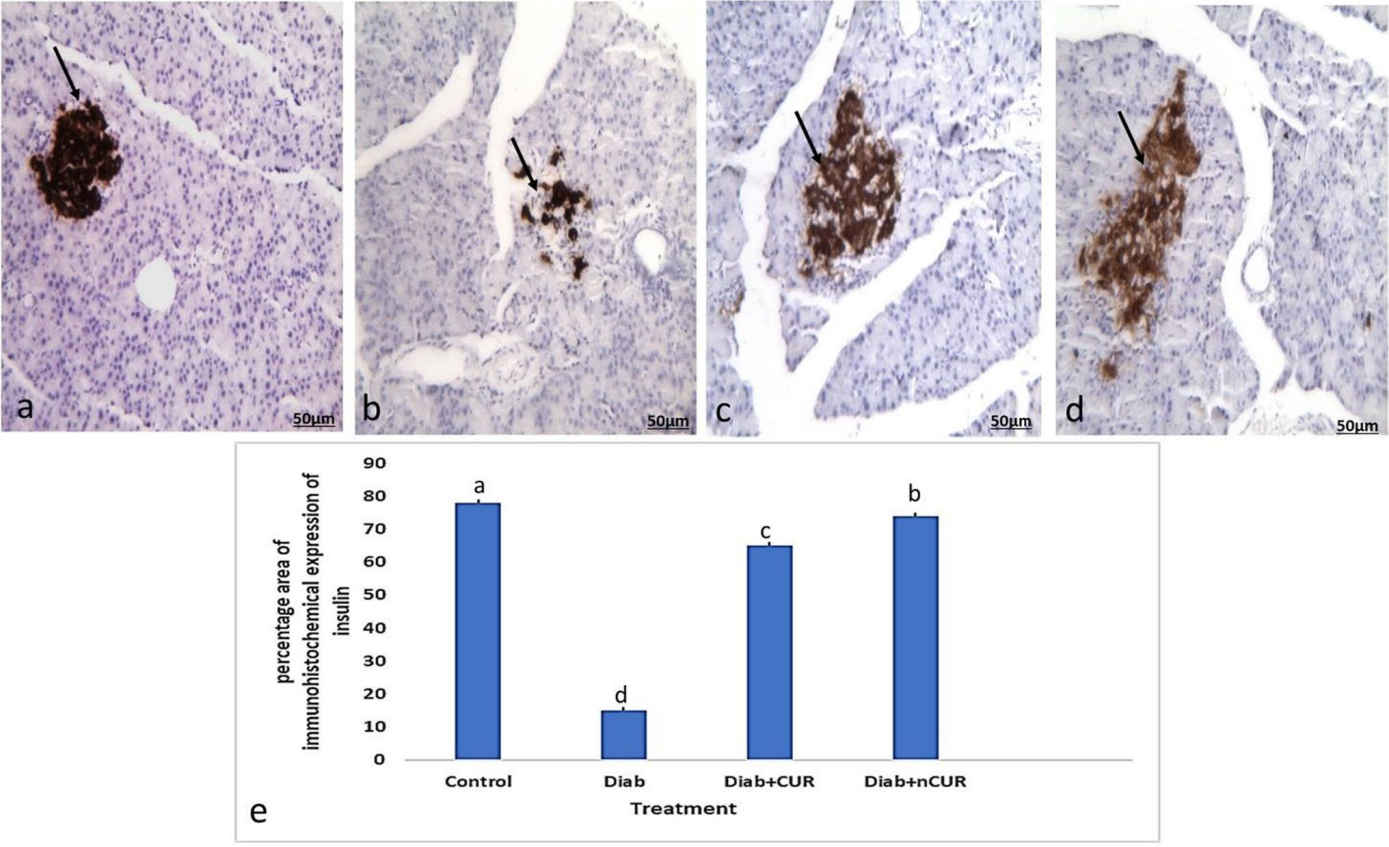
Photomicrographs of insulin immunohistochemical staining of pancreatic islets. **a** Section of the pancreas of the control group showed strong immunoreactivity of insulin in beta cells, which occupy most of the islet (arrow). **b** Pancreas of a Diab rat showed marked reduction in the immunohistochemical expression of insulin in beta cells (arrow). **c** The pancreas of Diab + CUR group showed apparent marked increase in immunoreactivity of insulin in beta cells in comparison with control group. **d** The pancreas of Diab + nCUR group showed apparent marked increase in immunoreactivity of insulin in beta cells in comparison with control group (IHC by anti-insulin antibodies, × 200). **e** Percentage area of insulin immunohistochemical staining of pancreatic islets. Statistical significance was evaluated by one-way ANOVA followed by Tukey test at *P* < 0.05. The mean values with different superscript letters on the bars were significantly different at *P* < 0.05. Diab, STZ-induced diabetic rats; Diab + CUR, STZ-induced diabetic rats that were given a daily oral dose of 100 mg/kg/day of curcumin were dissolved in carboxymethylcellulose (CMC) 1:10 (w/v); Diab + nCUR, STZ-induced diabetic rats that were given a daily oral dose of 100 mg/kg/day of nano-curcumin were dissolved in CMC 1:10 (w/v)

In the control group, insulin’s intracytoplasmic expression was strongly positive as evidenced by the presence of dark brown granules in the cytoplasm of beta cells shown in Fig. 4a. The beta cells’ cytoplasmic positive insulin expression was significantly reduced in the Diab group in Fig. 4b. Figure 4c, d shows that the immunostained β-cells from the Diab + CUR and Diab + nCUR groups resembled those from the control group, with a substantial (*P* < 0.05) increase in the insulin’s IHC staining area percentage in the Diab + nCUR group compared to the Diab + CUR group.

**Fig. 5:**
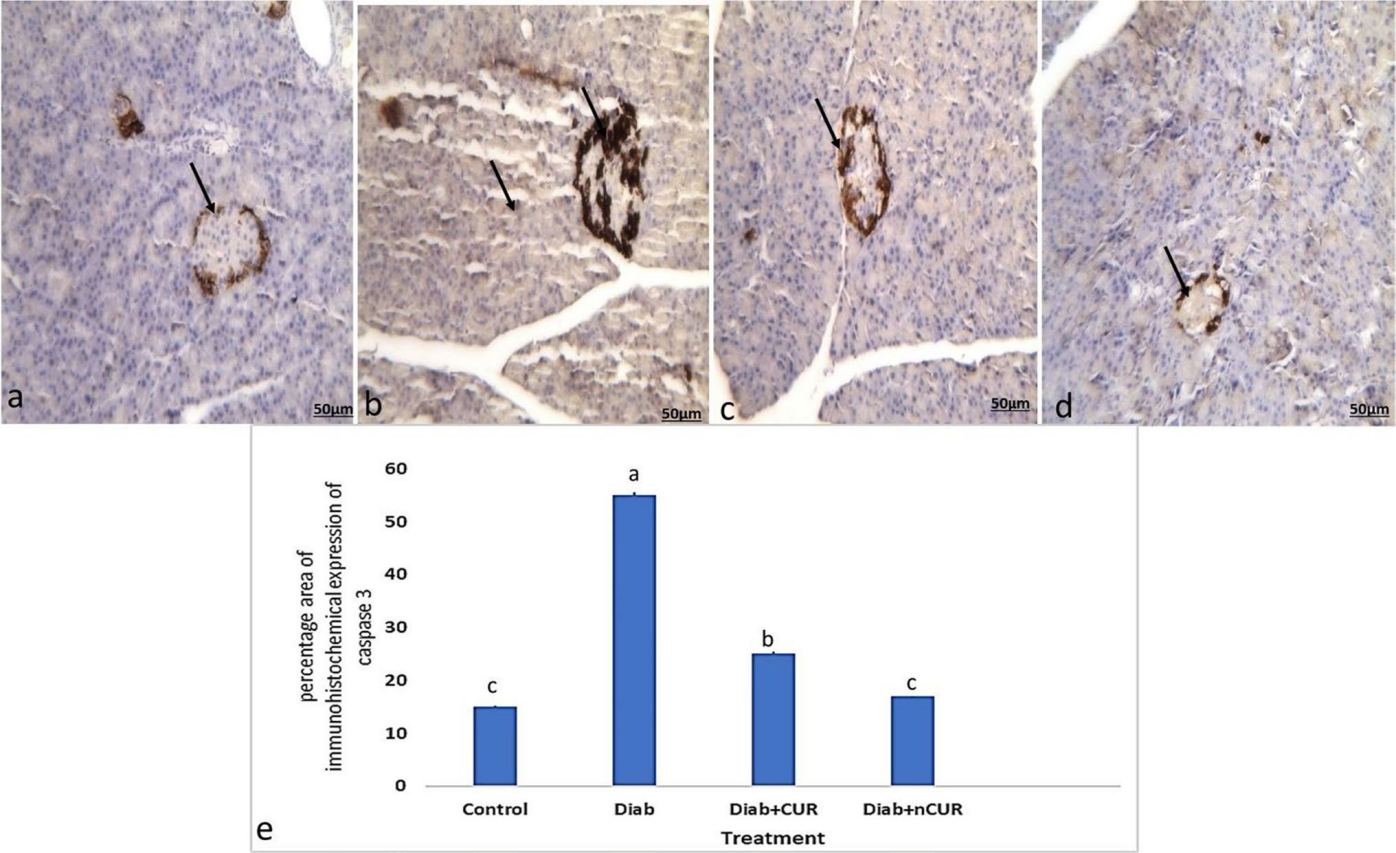
Photomicrographs of caspase-3 immunohistochemical staining of pancreatic islets and acinar. **a** Section of the pancreas of the control group showed weak immunoreactivity of caspase-3 in beta cells (arrow). **b** Pancreas of a Diab rat showed marked increase in the immunohistochemical expression of caspase-3 in beta and acinar cells (arrow). **c** The pancreas of Diab + CUR group showed apparent marked decrease in the immunoreactivity of caspase-3 in beta and acinar cells in comparison with control group. **d** The pancreas of Diab + nCUR group showed apparent marked decrease in the immunoreactivity of caspase-3 in beta and acinar cells in comparison with control group (IHC by anti-caspase 3 antibodies, × 200). **e** Percentage area of caspase-3 immunohistochemical staining of pancreatic islets. Statistical significance was evaluated by one-way ANOVA followed by Tukey test at *P* < 0.05. The mean values with different superscript letters within the same row are significantly different at *P* < 0.05. Diab, STZ-induced diabetic rats; Diab + CUR, STZ-induced diabetic rats that were given a daily oral dose of 100 mg/kg/day of curcumin were dissolved in carboxymethylcellulose (CMC) 1:10 (w/v); Diab + nCUR, STZ-induced diabetic rats that were given a daily oral dose of 100 mg/kg/day of nano-curcumin were dissolved in carboxymethylcellulose (CMC) 1:10 (w/v)

Beta and acinar cells in the control group had mildly positive immunohistochemical evidence of caspase-3 expression in the form of dark brown granules in the cytoplasm showed in Fig. 5a. In the cytoplasm of beta and acinar cells, the Diab group had a noticeably higher level of positive caspase-3 expression (Fig. 5b). In contrast to the Diab group, immunostained beta and acinar cells from Diab + CUR and Diab + nCUR showed only weakly positive caspase-3 expression with considerably (*P* 0.05) lower IHC-stained area % (Fig. 5c, d).

## Discussion

Diabetes mellitus, one of the most common endocrine disorders, affects the body’s ability to produce or utilize insulin (Mukhtar et al. 2020). Efforts collaborate to find bio-friendly, eco-friendly, relatively safe, and cost-effective remedy. With greater research being performed in the area of traditional medicine, plant-based medications have transitioned from being a niche approach to becoming more widely used (Rathore et al. 2020). Despite being utilized to treat and prevent a number of chronic conditions, including diabetes, several nutraceuticals have shown problems with bioavailability, solubility, and biodegradability (Moballegh Nasery et al. 2020). The present research investigated the efficiency of CUR and nCUR toward STZ-induced diabetic damage to the pancreas in male Sprague–Dawley rats.

According to the current findings, diabetic rats’ final body weights were significantly lower than those of the controls. This outcome was consistent with that of Sarkhail et al. (2007), who discovered that body weight loss in diabetic rats was directly related to the breakdown of structural proteins and fats. Administration of CUR and nCUR considerably reduced the weight loss brought on by diabetes.

The current findings were consistent with those of Moskal et al. (2021), who showed that CUR and nCUR can enhance muscular insulin resistance, glucose metabolism by boosting oxidation of fatty acid and glucose that alleviated muscular protein loss, and skeletal muscle insulin sensitivity. Curcumin has been shown by Canistro et al. (2021) to have a positive impact on body weight because it inhibits the Janus kinase enzyme, which has been shown to play a crucial role in the pathogenesis of obesity.

In the current study, diabetic rats had significantly higher glucose levels than controls, and their insulin levels were lower. Because STZ comprises a glucose molecule coupled to a highly reactive methyl nitrosourea moiety, it is believed to have cytotoxic effects on cells (Wu and Yan 2015). The main cause of the observed hypoinsulinemia, which manifested as hyperglycemia in the STZ-administered group, is thought to be the damage of β-cells (Shivavedi et al. 2019). Our results corroborated those of Hidaka et al. (2002), who discovered that STZ-treated rats had lower serum insulin levels than normal control rats when they have hyperglycemia. Diabetic rats treated with CUR and nCUR exhibited lower blood glucose levels and higher insulin levels than the Diab group, although nCUR significantly increased insulin levels compared to CUR. The current findings corroborated those of Ganugula et al. (2017) and Shamsi-Goushki et al. (2020), who showed that therapy with CUR and nCUR, respectively, significantly improved insulin levels, lowered glucose levels, and prevented the death of pancreatic beta cells. According to El-Moselhy et al. (2011), CUR has been demonstrated to increase insulin sensitivity in the muscle tissue of insulin-resistant rodents by enhancing GLUT4 translocation from intracellular compartments to the plasma membrane. This results in an increase in cellular glucose uptake. By raising adiponectin levels and lowering leptin, CUR can reduce insulin resistance (Panahi et al. 2016). Additionally, CUR may increase insulin secretion by inducing β-cell activity (Rouse et al. 2014).

As clarified in the current study, STZ-induced decreased HDL-c and increased TC, TG, and LDL-c of the diabetic group. The observed dyslipidemia was parallel to that obtained by Wang et al. (2013) while contradictory with of Zubaidah et al. (2018) who found that total serum TC, LDL, TG, and HDL did not change in diabetic rat model. Diabetic dyslipidemia develops as a result of insulin insufficiency and hyperglycemia, which enhances lipolysis and fatty acids release from adipose tissue into circulation with a variation in their metabolism (Goldberg 2001).

When compared to diabetic non-treated rats, administration of CUR and nCUR to the rats caused an increase in HDL-c level and a decrease in TC, TG, and LDL-c levels. Neerati et al. (2014) investigated whether CUR capsule administration might improve blood lipid levels. Their findings demonstrated that compared to diabetic rats, the CUR-treated group had reduced levels of TC, LDL, VLDL, and TG and increased levels of HDL. According to Shamsi-Goushki et al. (2020), patients who received nCUR supplementation (80 mg/day) demonstrated a statistically significant decrease in FBS, TG, TC, and LDL compared to the placebo group. CUR is an antioxidant molecule, so it may lessen lipid-mediated oxidative and ER stress by either directly scavenging free radicals chemically or by activating antioxidant enzymes. Depending on the circumstances and solvents employed, the H-atom donation from CUR’s phenolic hydroxyl group was thought to be the source of the compound’s chemical reactive radical scavenging and antioxidant action (Škrovánková et al. 2012).

Streptozotocin increased pancreatic lipid peroxidation via elevating MDA contents than normal group. Higher quantities of MDA show that oxidative stress is linked to an increase in lipid peroxidation, which is involved in the pathogenesis of many diseases (Abuja and Albertini 2001). On the parallel side, the SOD and GSH levels in the Diab group were considerably lower than those in the control group. SOD is a crucial component of the body’s defense against oxygen. It facilitated the conversion of the O_2_ radical to oxygen or hydrogen peroxide (H_2_O_2_) and then to H_2_O (Zulaikhah 2017). In mammalian tissues, GSH is the most prevalent non-protein thiol at millimolar quantities. It functions as a regulator of cellular redox status and an essential intracellular antioxidant, shielding cells from harm brought on by lipid peroxides, reactive oxygen and nitrogen species, and xenobiotics (Kennedy et al. 2020). Strong evidence for the oxidative stress caused by STZ in pancreatic tissue is the depletion of SOD and GSH. The damage that was seen in the pancreatic histological sections was proof of this oxidative stress. According to Cheng et al. (2014), STZ’s capacity to produce oxygen free radicals led to an increase in MDA levels and a decrease in antioxidant enzyme levels in the pancreas.

SOD and GSH levels were raised in diabetic rats after CUR and nCUR administration. Compared to the CUR, nCUR dramatically raised SOD and GSH levels. On the parallel side, MDA levels in the Diab + CUR and Diab + nCUR groups dramatically decreased as compared to the Diab group. These findings were consistent with Sharma et al. (2006), who found that diabetic rats had significantly higher levels of lipid peroxidation as seen by markedly higher MDA and lower GSH levels as compared to control rats given with a vehicle treatment. In comparison to diabetic rats, chronic treatment with curcumin (15 and 30 mg/kg per day) dramatically corrected the elevated lipid peroxidation and decreased GSH.

The effects of CUR and nCUR on many biochemical and physiological processes have been the focus of numerous studies, although there are few experimental studies addressing the histological alterations and immunohistochemical expression to the islets of Langerhans in diabetes. Hyperglycemia, a reduction in insulin, and the loss of the beta cells’ microscopic structure in the pancreatic islets of Langerhans make up the global problem of diabetes mellitus (Abunasef et al. 2014b). The results of earlier biochemical, histological, and immunohistochemical research were identical to those of the present study. According to Abdel-Mageid et al. (2018), the STZ-induced rise of fasting plasma glucose concentrations and pancreatic insulin content was reversed by CUR and nCUR. It was proposed that CUR and nCUR would guard beta cells from the toxicity of STZ (Shamsi-Goushki et al. 2020). The current biochemical findings are consistent with the endocrine pancreas’ histology and immunohistochemical findings. In diabetic rats, the insulin-producing beta cells were destroyed and decreased in number, which led to a decrease in insulin immunohistochemistry expression and an increase in caspase-3 immunohistochemical expression.

Although STZ’s degenerative effects on the pancreatic islets were reversed by CUR and nCUR. In diabetic rats, they were able to lessen the islets of Langerhans morphological changes. The results reported by Adeghate and Ponery (2001) who discovered a lower number of insulin immunoreactive cells in the pancreatic islets of diabetic rats are supported by the immunohistochemistry findings and the morphometric measurements presented in our work. The current findings demonstrated that CUR and nCUR can significantly enhance beta cell function, as demonstrated by the increased insulin level and their immunohistochemistry expression. Additionally, it has been suggested that CUR and nCUR can cure pancreatic beta cell damage from free radicals produced by oxidative stress and prevent membrane disruption.

The current finding showed that diabetic rats had significantly lower PDX1 expression than the control group. The administration of CUR and nCUR exhibited significant increment in the fold change of PDX1 expression compared to diabetic rats with more pronounced promotion in nCUR-treated group than the CUR group. This donated more efficacy of nCUR than CUR in amelioration PDX1 reduction. Such amelioration could enhance the insulin producing machinery cascade as noted by the improved insulin level in nCUR group and CUR with more pronounced effect of nCUR. Briefly, the administered dose of CUR and nCUR may play a therapeutic function in reversing the biochemical and microscopic alterations of pancreatic beta cells in STZ-induced diabetic rats, according to the study’s findings. To the best of our knowledge, the herein study is the first study that handles the influence of CUR and nCUR on PDX1 expression in diabetic animal model with emphasis on insulin protein expression as detected by immunohistochemistry as well as apoptotic protein caspase-3.

## Conclusion

The administration of CUR and nCUR could induce a therapeutic potential against STZ-induced diabetes in male Sprague–Dawley rats. They exerted their effect via amelioration of STZ-induced oxidative stress, improving FBS, insulin level and immunoreactivity, lipid profile, and reducing islet apoptosis as evidenced by their caspase-3 immunoractivity reduction. The usage of nCUR exerted more superior effect in promotion of serum insulin level, insulin pancreatic immunoreactivity, SOD, GSH and PDX1 expression and reduction of pancreatic caspase-3 than CUR.

## Data Availability

The datasets generated during and/or analyzed during the current study are available from the corresponding author on reasonable request.

## Declarations

### Disclaimer

The authors declare that all data were generated in-house and that no paper mill was used.
